# Detection of *Coxiella burnetii* and equine herpesvirus 1, but not *Leptospira* spp. or *Toxoplasma gondii*, in cases of equine abortion in Australia - a 25 year retrospective study

**DOI:** 10.1371/journal.pone.0233100

**Published:** 2020-05-26

**Authors:** Rumana Akter, Alistair Legione, Fiona M. Sansom, Charles M. El-Hage, Carol A. Hartley, James R. Gilkerson, Joanne M. Devlin

**Affiliations:** 1 The Melbourne Veterinary School, The University of Melbourne, Parkville, Victoria, Australia; 2 Department of Medicine, The University of Melbourne, Parkville, Victoria, Australia; University of Kentucky, UNITED STATES

## Abstract

Equine abortion is a cause of severe economic loss to the equine industry. Equine herpesvirus 1 is considered a primary cause of infectious abortion in horses, however other infectious agents can also cause abortion. Abortions due to zoonotic pathogens have implications for both human and animal health. We determined the prevalence of *Coxiella burnetii*, *Leptospira* spp. and *Toxoplasma gondii* in 600 aborted equine foetal tissues that were submitted to our diagnostic laboratories at the University of Melbourne from 1994 to 2019. Using qPCR we found that the prevalence of *C*. *burnetii* was 4%. The highest annual incidence of *C*. *burnetii* was observed between 1997–2003 and 2016–2018. The prevalence of *C*. *burnetii* in Victoria and New South Wales was 3% and 6% respectively. All the samples tested negative for *Leptospira* spp. and *Toxoplasma gondii* DNA. Equine herpesvirus 1 DNA was detected at a prevalence of 3%. This study has provided evidence for the presence of *C*. *burnetii* in equine aborted foetal tissues in Australia, but the role of *C*. *burnetii* as potential cause of abortion in Australia requires further investigation. *C*. *burnetii* is a zoonotic disease agent that causes the disease ‘Q fever’ in humans. We recommend that appropriate protective measures should be considered when handling material associated with equine abortions to reduce the risk of becoming infected with *C*. *burnetii*.

## Introduction

Abortion in horses, classified as loss of the foetus before 300 days of gestation, causes severe economic loss to the equine industry [1, 2]. Several previous studies have investigated the causes of equine reproductive loss globally and have identified that losses due to infectious, non-infectious and idiopathic causes were 20–60%, 36–72% and 8–16.0% respectively [1, 3–5]. Although equine alphaherpesvirus-1 (EHV-1) is considered a major cause of infectious equine abortion, recent studies have identified bacteria as additional important infectious causes of abortion. The bacteria identified in these studies include *Streptococcus* species [1, 4–6] as well as *Chlamydia psittaci*, which has recently been identified as an important zoonotic cause of equine abortion in Australia [7]. Other zoonotic pathogens such as *Coxiella burnetii*, *Leptospira* spp. and *Toxoplasma gondii* are known to cause abortion in multiple animal species such as cattle, sheep and goats but are less commonly reported in horses where they have been associated with only sporadic cases to date [8–10].

*Coxiella burnetii* is an intracellular gram-negative bacterium that causes a zoonotic disease known as coxiellosis in humans and animals. The disease is frequently termed ‘Q fever’ in humans. It has been detected in a broad range of animal hosts including domestic and wild mammals, birds, as well as in arthropods such as ticks [11, 12]. Infection can range from asymptomatic to severe in humans. Acute forms of diseases in humans are characterized by a non-specific flu-like syndrome, pneumonia, or hepatitis. Chronic forms of the disease can be manifested by endocarditis and chronic hepatitis [13–15]. Infected animals may not show clinical signs of disease, or may develop reproductive disorders, abortions and stillbirths or delivery of weak neonates [16, 17]. Depression, fever, enteritis, and bronchopneumonia have been reported in experimentally infected horses [18].

*C*. *burnetii* has been detected in people who ride horses or visit horse facilities, but contact with other livestock or ticks at the horse facilities have often been considered to be the source of infection [19–21]. Although human infection transmitted from horses has not been reported, studies have hypothesised that individuals such as equine veterinarians or breeders could potentially be at higher risk of infection [22–25]. The role of horses as a reservoir of Q fever is unclear. Few serological studies are available, but those that have been undertaken have reported seroprevalence ranging from 0–52.5% [20, 26–31]. Moreover, some studies have detected *C*. *burnetii* in equine aborted foetuses or placentas, but the association of *C*. *burnetii* with reproductive losses are unclear [4, 20, 21] despite several epidemiological studies investigating the role of *C*. *burnetii* infection in equine abortion cases in Europe [4, 20, 26]. The role of *C*. *burnetii* as a possible cause of equine abortion in Australia has not been investigated.

*Leptospira* spp. are the causative agent of the zoonotic disease leptospirosis. The pathogenic species of *Leptospira* cause leptospirosis in a wide variety of hosts [32] including domestic animals (including cattle, sheep, pigs, horses and dogs), wildlife and humans [33, 34]. *Leptospira* transmits by direct or indirect contact with the infected host such as ingestion of contaminated food and inhalation of urine droplets from infected hosts [35]. Infection in humans varies from mild to fatal and fever is a common clinical sign of infection in the acute phase of disease, while headaches, chills and myalgia are non-specific signs of leptospirosis [36, 37]. Leptospirosis in animals can vary with the infecting species or serovar of *Leptospira* and is well defined for many livestock species including cattle, pigs and small ruminants [38, 39]. In horses, recurrent uveitis and periodic ophthalmia are the most common clinical manifestations [36]. *Leptospira* has also been detected in cases of stillbirths, abortions, weak foals, and hepatic and renal dysfunction in horses [40–44]. Serological studies indicate that horses are frequently exposed to *Leptospira* and the seroprevalence can range from 2–79% [45–50]. Recently, *Leptospira* has detected in equine aborted material by PCR in Brazil [42] and the USA [51]. In Australia, several serological surveys have detected *Leptospira* in healthy horses [52–55] but the occurrence of these bacteria in abortion cases are unknown.

*T*. *gondii* is a ubiquitous, obligate intracellular protozoan parasite. It causes the zoonotic disease toxoplasmosis in humans and all warm-blooded animals [56–58] and results in substantial financial loss to the livestock industry by causing reproductive loss and mortality [59, 60]. In horses, *T*. *gondii* infection is frequently subclinical. However, fever, ataxia, retinal degeneration and encephalomyelitis, as well as abortion or stillbirth in pregnant mares, has been observed [61, 62]. The epidemiology of *T*. *gondii* infection in horses has been investigated in several countries. Those studies reported that seroprevalence of *T*. *gondii* in horses may range from 0–100% [63–66]. *T*. *gondii-*like protozoa have also been identified in an equine aborted foetus by histopathology with associated pathological changes, indicating that *Toxoplasma* could play a role in equine abortion [67]. Some studies have explored the presence of *T*. *gondii* in Australian livestock, particularly in sheep and cattle, [68, 69] but its presence in Australian horses has not been investigated.

This study aimed to determine the prevalence of these three pathogens (*Coxiella*, *Leptospira and Toxoplasma)* in equine abortion cases in Australia using archived samples extending over 25 years (2004 to 2019). The prevalence of EHV-1 was also assessed.

## Materials and methods

### Sample collection

This study used archived samples from equine abortion cases that were submitted to our diagnostic laboratories at the University of Melbourne from 1994 to 2019. The samples were predominantly from New South Wales (NSW), and Victoria (VIC), Australia (Table 1). For each case, different types of foetal tissue including lung, liver, spleen, placenta and thymus were submitted. Based on the availability of tissues, a total of 600 foetal tissues (lung, spleen, thymus and placenta for each foetus) were selected from the archive for this study. The tissues were stored at -80°C in 1.5 mL tubes after submission. Selected samples were thawed, and a plastic-shafted rayon tipped swab (Copan Italia) was used to sample each tissue. Swabs from tissue originating from the same foetus were combined in 500 μL of PBS and the pooled swabs were stored at -80°C until DNA extraction.

**Table 1:** Number of selected foetal tissues submitted between 1994–2019.

State	Number of submitted samples (N)
**Victoria**	395
**New South Wales**	182
**Queensland**	4
**Tasmania**	1
**South Australia**	13
**Northern territory**	1
**Western Australia**	4
**Total**	600

### DNA extraction

Each tube of swab/PBS solution was vortexed for approximately 5 sec before a 200 μL aliquot was removed for extraction. DNA was extracted from the PBS (pH 7.4) solution by a Kingfisher robot with a MagMAX^™^ Core Nucleic Acid Purification Kit (Thermo Fisher Scientific) according to the manufacturer instructions. Axenically cultured *C*. *burnetii* Nine Mile Phase Ⅱ was used as a positive extraction control and PBS was used as a negative extraction control. Extracted DNA was eluted in 90 μL of elution buffer and stored at -80°C for further use. These extraction methods were followed for extraction of 483 swab/PBS pools encompassing samples collected between 1994–2004. The remaining 117 samples submitted to our diagnostic laboratories at the University of Melbourne or to Agriculture Victoria between 2010–2019 had been pre-extracted from pooled tissue swab for routine diagnostic testing and stored prior to this study.

### Polymerase Chain Reaction (PCR)

#### Real-time qPCR for detection of *C*. *burnetii*

Real-time qPCR targeting an 82 bp region of the *ompA* gene was performed to detect *C*. *burnetii* DNA [70]. A total of 20 μL reaction mixture comprising of 10 μL of Sensifast SYBR No Rox master mix (Bioline), 0.4 μM of each primer (S1 Table) (Integrated DNA Technologies), 5 μL of template DNA template, and Milli-Q filtered water to reach the final volume were prepared. The cycling parameters were; 3 mins at 95°C, followed by 45 cycles of 5 sec at 95°C and 20 sec at 65°C. Genomic DNA extracted from *C*. *burnetii* Nine Mile Phase Ⅱ was used as positive control template and Milli-Q filtered water was used as negative control template.

#### TaqMan qPCR amplification to detect pathogenic *Leptospira*

Pooled swabs were screened to detect a 242 bp fragment of the *lipL32* gene of pathogenic *Leptospira* by using TaqMan qPCR as described previously [71]. Briefly, a 20 μL PCR reaction mixture comprising of 10 μL of TaqMan Universal PCR master mix (Thermo Fisher Scientific), 0.7 μM of each primer (S1 Table), 0.15 μM of probe (S1 Table) (Bio Search Technologies), 5 μL of template DNA and Milli-Q filtered water to reach the final volume. The cycling conditions were; 10 min at 95°C, followed by 45 cycles of amplification at 95°C for 15 sec and 58°C for 60 sec. A commercially produced (Integrated DNA Technologies) plasmid containing the full *lipl32* gene was used as positive control template. Milli-Q filtered water was used as a negative control template.

#### Real-time qPCR for detection of *T*. *gondii*

Extracted genomic DNA was used in a qPCR to amplify a 529 bp highly repetitive element as described previously [72]. The 20 μL reaction was comprised of 10 μL of GoTaq qPCR Master Mix (Promega), 0.15 μM of the probe (S1 Table), 0.15 μM of each primer (S1 Table) (Integrated DNA Technologies), 5 μL of template DNA and Milli-Q filtered water to reach final reaction volume. The reaction mixture was subjected to an initial incubation of 95°C for 2 minutes, and then 45 cycles of denaturation at 95°C for 15 sec and annealing/extension at 60°C for 60 sec. Laboratory stock of genomic DNA of *T*. *gondii* was used as positive control template and Milli-Q filtered water was used as a negative control template.

### Limit of detection

The limit of detection of each qPCR assay (Table 2) was determined by testing ten-fold serial dilutions of 1×10^9^ or 1×10^8^ copies of genomic/ plasmid DNA in triplicate. All qPCRs were carried out using an Mx3000P qPCR (Agilent Technologies) thermocycler. The cycle threshold value (Ct value) was used to determine positive *Coxiella*, *Leptospira* and *Toxoplasma* DNA with potentially positive PCR products visualised by UV transillumination using Bio-Rad Image Lab software after electrophoresis of 10 μL aliquots through a 2% agarose gel containing SYBR Safe DNA gel stain and run in TBE buffer (45 mM Tris-HCl, 45 mM Boric acid, 1mM EDTA, pH 8.3) at 90 V. Hyper ladder 25 and 100 bp (Bioline) was used for the estimation of amplicon size.

**Table 2:** Details of the assays used in this study.

Pathogen	Type of assay	Product size (bp)	Cut-off Ct value	Limit of detection (Copy number /reaction)
***C*. *burnetii***	Real-time qPCR	82	38	20
***Leptospira* spp.**	TaqMan qPCR	242	36	200
***T*. *gondii***	Real-time qPCR	529	37	200
***Equine herpesviruses***	Nested conventional PCR	215	Not applicable	≈ 200*

* The PCR to detect equine herpesviruses is a non-quantitative nested PCR that can detect all herpesviruses. The limit of detection for another alphaherpesvirus (infectious laryngotracheitis virus) has previously been estimated at approximately 200 copies/reaction [73].

### Quantitation of *C*. *burnetii* loads from equine foetal tissues

Genome copy numbers of *C*. *burnetii -*positive samples were calculated using a standard curve consisting of 10-fold dilutions, in triplicate, of a purified plasmid containing *C*. *burnetii ompA* at 10^7^ to 10^1^ copies per reaction. A Qubit 3.0 fluorometer (Invitrogen) was used to calculate the copy numbers of plasmid DNA.

### Detection of herpesviruses

A universal PCR targeting the herpesvirus DNA polymerase gene was carried out in a nested format with previously published primers (S1 Table) [74]. In the first round of PCR, the total volume of each reaction was 50 μL which contained 2 mM MgCl_2_ (Promega)_,_ 0.2 mM of each dNTPs (Bioline), 0.2 μM of each primer (S1 Table), 1× Green GoTaq Flexi Buffer (Promega), 1 U of GoTaq DNA polymerase (Promega), 5 μL of template DNA, and Milli-Q filtered water to reach the final volume. DNA extracted from laboratory stocks of EHV-1 was used as a positive control template and Milli-Q filtered water was used as a negative control template. The reaction mixtures were subjected to the following thermocycling parameters: initial denaturation at 95°C for 5 min followed by 45 cycles of denaturation at 95°C for 30 sec, annealing at 46°C for 60 sec, and extension at 72°C for 1.5 min, followed by a final extension of 72°C for 5 min.

In the second-round PCR, the volume and concentration of all reagents were the same as the first round except that the concentration of primer was increased to1 μM. Cycling conditions were also the same except that the annealing and extension times were reduced to 30 and 60 sec respectively. All PCRs were carried out in a T100 Bio-Rad thermal cycler. The amplified PCR products were visualised by UV transillumination using Bio-Rad Image Lab software after electrophoresis of 10 μL aliquots through a 2% w/v agarose gel containing SYBR Safe DNA gel stain in TBE buffer at 90 V. Hyperladder 25 bp (Bioline) was used for the estimation of the amplicon size. Since this PCR amplifies equine alphaherpesviruses-1, -3 and -4, as well as the equine gammaherpesviruses-2 and -5, amplicons of the expected sizes were sequenced to identify the virus type. Then, the identified herpesvirus (EHV-1) were further analysed using ORF30 and ORF68 PCR.

### EHV-1 Open Reading Frame 30 (ORF30) and 68 (ORF68) PCR

In the samples identified as EHV-1 positive by the universal herpesvirus PCR, the EHV-1 ORF30 gene was amplified using nested PCR as described previously to determine if the isolates were the non-neuropathogenic or neuropathogenic genotype [75]. In the first round of PCR, the total volume of each reaction was 50 μL which contained 2 mM MgSO_4_ (Invitrogen), 0.2 mM of each dNTPs (Bioline), 0.4 μM of each primer (S1 Table), Platinum Taq DNA polymerase High Fidelity Buffer (Invitrogen), 1U of Platinum Taq DNA polymerase High Fidelity (Invitrogen), 10 μL of template DNA, and Milli-Q filtered water to reach the final volume. DNA extracted from laboratory stocks of EHV-1 was used as positive control template and Milli-Q filtered water was used as a negative control template. The cycle conditions were: 1 cycle of 94 ˚C for 30 sec, followed by 35 cycles of 94 ˚C for 30 sec, 48˚C for 30 sec and 68˚C for 60 sec. In the second-round, 10 μL of PCR product from the first round was used as a template. The volume and concentration of all reagents were the same as the first round. Cycling conditions were also the same except that extension times were reduced to 60 sec.

The ORF68 gene was also sequenced in the current study. A region of this gene has sometimes been considered as a marker that can be suitable for epidemiological studies. EHV-1 ORF68 gene was amplified using conventional PCR as described previously [76]. EHV-1 ORF68 was amplified with 1.25 U of GoTaq DNA polymerase (Promega) in a 50 μL reaction mixture containing 1.5 mM MgCl_2_ (Promega), Green GoTaq Flexi Buffer (Promega), 0.2 mM of each dNTPs (Bioline), PCR Enhancer solution (Invitrogen), 0.4 mM of each primer (S1 Table), 5 μL of template DNA and Milli-Q filtered water to reach the final volume. DNA extracted from laboratory stocks of EHV-1 was used as positive control template and Milli-Q filtered water was used as a negative control template. All PCR reactions were carried out in a T100 Bio-Rad thermal cycler. The cycle conditions were: 1 cycle of 95 ˚C for 3 min, followed by 40 cycles of 95 ˚C for 60 sec, 56 ˚C for 60 sec and 72 ˚C for 120 sec. The amplified PCR products were visualised by UV transillumination using Bio-Rad Image Lab software after electrophoresis of 10 μL aliquots through a 2% agarose gel containing SYBR Safe DNA gel stain in TBE buffer at 90 V. Hyperladder 1kb (Bioline) was used for the estimation of the amplicon size.

### Sequencing of PCR products

PCR products were purified from the PCR reaction mixtures using the QIAquick Gel Extraction Kit (Qiagen) according to the manufacturer’s instruction and eluted in 30 μL elution buffer (10 mM Tris-Cl, pH 8.5). The quantity of purified DNA was estimated using a spectrophotometer (NanoDrop Technologies) and sequenced using Big Dye Terminator (BDT) v3.1 (Applied Biosystems) according to the manufacturer’s instructions. The products were sent to the Australian Genome Research Facility (AGRF) for sequencing. Geneious bioinformatics software version 11.1.4 (Biomatters Ltd) was used to trim, edit and align all obtained sequences. Nucleotide sequences were compared with publicly available sequences in the Genbank database (National Center for Biotechnology Information), (https://www.ncbi.nlm.nih.gov/genbank) using the NCBI Nucleotide Basic Local Alignment Search Tool (BLASTN) online algorithm (https://blast.ncbi.nlm.nih.gov/Blast.cgi).

### Statistical analysis

The statistical significance of the prevalence of target pathogen between different states was tested using Fisher’s exact test in IBM SPSS software. The result was considered statistically significant if the *P* value was < 0.05. The percentage of positive cases for each year of the study was calculated using the number of samples tested in each year and the number of samples that were positive for the same year.

## Results

### Prevalence of potential pathogens in foetal tissues

**Fig 1:**
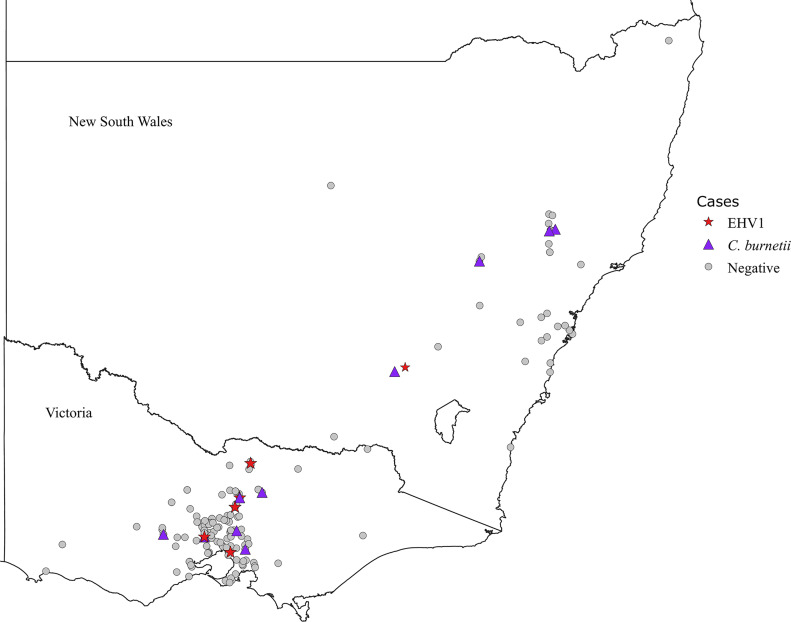
Map of state of Victoria and New South Wales, Australia showing the location of positive isolates. Here, positive isolates and negative isolates in VIC and NSW are represented by different symbols and colours. The state map is reprinted from an outline map of Australia (Geoscience Australia, Canberra) under a CC BY license with permission from the **Commonwealth of Australia (Geoscience Australia)**. Original copyright (2005).

The Ct cut‐off value for each pathogen was determined using a standard curve of control samples containing known concentrations of *C*. *burnetii*, *Leptospira* and *T*. *gondii* target DNA. DNA from these positive controls were consistently detected at Ct values of 38, 36 and 37, respectively. This corresponsed to limits of of detection of 20, 200 and 200 copy numbers per reaction, respectively (Table 2). Thus test samples were considered to be *C*. *burnetii*, *Leptospira* spp. and *T*. *gondii* positive if the Ct values were lower than these values, and if the size of DNA product was consistent with the positive control sample. In the present study, *C*. *burnetii* was detected in 21 abortion cases giving a prevalence of 4% (95% CI: 2–5%) (Table 3). Of the 21 positive isolates, 10 cases (3%, 95% CI: 1–5%) were from Victoria and 11 cases (6%, 95% CI: 3–11%) were from NSW (Fig 1). There was no significant difference observed between the prevalence of *C*. *burnetii* in Victoria and NSW. The annual incidence of *C*. *burnetii* ranged from 0–14% and was highest between 1997–2003 and 2016–2018 (Table 4). A selection of positive samples were sequenced to confirm that the amplified products were *C*. *burnetii* DNA. The sequences were compared to those available in Genbank. The nucleotide sequence of the amplified *ompA* gene had 98.4–100% nucleotide identity to the reference sequences AY502769.1 and MK168663.1.

**Table 3:** Prevalence of potential pathogens in equine abortion cases.

Pathogen	Positive (N)	Prevalence (%)	95% CI (%)	Negative (N)	Total (N)
*C*. *burnetii*	21	4	2–5	579	600
*Leptospira* spp.	0	0	0–1	600	600
*T*. *gondii*	0	0	0–1	600	600
EHV-1	18	3	2–5	579	600

N = Number, CI = Confidence interval

**Table 4:** Number of *C*. *burnetii* positive cases between 1994–2019.

Years	Samples tested	Positive (N)	Positive (%)	Negative (N)
1994	5	0	0	5
1995	7	0	0	7
1996	4	0	0	4
1997	32	4	13	28
1998	32	2	6	30
1999	62	2	3	60
2000	54	0	0	54
2001	64	2	3	62
2002	86	1	1	85
2003	90	5	6	85
2004	47	0	0	47
2010	2	0	0	2
2011	17	0	0	17
2012	13	0	0	13
2013	14	0	0	14
2014	9	0	0	9
2015	21	0	0	21
2016	21	3	14	18
2017	8	1	13	7
2018	9	1	11	8
2019	3	0	0	3
Total	600	21	3.5	579

No samples were positive for *Leptospira* or *Toxoplasma* spp.(Table 3). The samples were also tested for herpesviruses and 18 samples were identified as herpesvirus positive. All of the detected herpesvirus were EHV-1. The prevalence of EHV-1 was 3% (Table 3). One sample was positive for both *C*. *burnetii* and EHV-1.

### Load of *C*. *burnetii* in positive samples

Genome copy numbers of *C*. *burnetii* in the positive samples were quantified by *ompA* qPCR. The median (range) of *C*. *burnetii* in the positive samples was 3.77 × 10^2^ (4.03 × 10^1^–2.6 × 10^3^) genome copies per reaction.

### EHV-1 ORF30 and ORF68 sequence analysis

**Fig 2:**
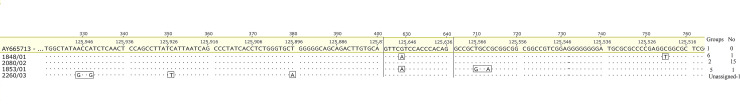
Sequences of EHV-1 ORF68 are aligned with reference sequence (GenBank AY665713.1). The representatives of variation in sequences in archived isolates are shown and the position of nucleotide relative to Ab4 (GenBank AY665713.1) are numbered. The sequence identity is denoted by dots and non-synonymous changes are highlighted.

Sequence analysis of the ORF30 gene showed that all EHV-1 detected from the equine abortion cases were of the non-neuropathogenic genotype (A_2254_). The nucleotide 125,387–125,945 region of ORF68 gene has sometimes been considered a useful marker related to the geographical origins of EHV-1 isolates [75]. A series of SNPs were used to group the isolates based on ORF68 sequence. Based on the previously proposed classification, 15 isolates were classified as group 2, and two of the isolates were classified as either group 5 or 6. One isolate could not be placed into a classified group (Fig 2) [75] and has submitted in GenBank under the accession number MN786807.

## Discussion

The current study investigated the prevalence of the important zoonotic pathogens *C*. *burnetii*, *T*. *gondii* and pathogenic *Leptospira* spp. in equine aborted foetal tissues. The distribution of two of these pathogens in equine foetal tissues has been described previously. *C*. *burnetii* has been detected in high levels in equine foetal lung and placenta [4]. Leptospira has been detected in equine foetal lung, spleen and kidney [51]. The distribution of *T*. *gondii* in equine aborted material is not as well described [77]. In this study we pooled available foetal tissues from each abortion event in order to increase the likelihood of including tissue that contained a high pathogen load.

*C*. *burnetii* was detected by qPCR for the first time in equine aborted foetal tissues in Australia. The prevalence of *C*. *burnetii* in equine abortion cases was 4% which is consistent with previous international studies that have reported prevalence rates of 4% (1/23) in aborted equine foetuses using real-time PCR in Germany in 2012 [21] and 3.4% (22/629) and 1.5% (6/407) using PCR in France in 2009 and 2012, respectively, [4, 78]. A similar study reported that the prevalence of *C*. *burnetii* was 8% in abortion cases in the Netherlands in 2011 [20].

In the present study, low loads of *C*. *burnetii* were detected in the equine foetal tissues when compared to those reported in ovine and caprine placental tissues [79]. This is consistent with a previous study that also reported low loads of *C*. *burnetii* DNA in equine placental tissues [20]. Detection of *C*. *burnetii* DNA is not sufficient in itself to implicate the bacteria as the cause of abortion [4]. Indeed, the low loads of *C*. *burnetii* DNA detected in equine foetal tissues could indicate that the bacteria may not be the cause of the abortion event. Future work to determine the prevalence and load of *C*. *burnetii* infection in animals with normal reproductive outcomes would be important to understand the significance of infection in horses [26]. Moreover, studies to determine if the presence of *C*. *burnetii* DNA is associated with pathological changes are indicated [80] as we did not have histological examination results for any of the samples used in this current study. The low loads of *C*. *burnetii* DNA present in the samples in this current study prevented genotyping being performed successfully using genotyping systems described previously [81, 82], consistent with a previous equine study [20].

*Leptospira* spp. was not detected in any cases of abortion in this current study. Previous studies have used PCR to detect *Leptospira* DNA in 12 aborted equine foetal tissues or fluids in USA and Brazil [42, 51]. However a large study performed in the USA by Tengelsen et al. (1997) reported that 290 equine foetal tissues were *Leptospira* negative by direct immunofluorescence [83]. Serological testing has revealed a high *Leptospira* seroprevalence in clinically normal horses in Queensland, Australia [52] but no previous studies have investigated *Leptospira* infection in equine abortion cases in Australia. The negative results of the current study suggest that *Leptospira* spp. may not be associated with equine abortion in Australia, although it would be beneficial for future studies to incorporate higher numbers of samples from other geographical regions, including Queensland, as the samples in this current study were mostly from Victoria and NSW.

*T*. *gondii* DNA was not detected in equine foetal tissues in the current study. This result is consistent with results from a study conducted in Hungary between 1998–2000, where all 96 equine aborted foetal tissues were *T*. *gondii* negative by immunohistochemistry [6]. This finding is despite previous studies which have shown that *T*. *gondii* is able to cross the equine placenta in an experimental model, and the reported detection of *T*. *gondii-*like protozoa in an equine aborted foetus [67, 84]. Serological surveys suggest that subclinical *Toxoplasma* infection is common among horse populations worldwide [64, 85, 86] although it should be noted that no such studies have been conducted in Australia. Taken together these results suggest that that *T*. *gondii* is not associated with abortion in horses in Australia.

Equine herpesvirus-1 is recognised as an important cause of equine infectious abortion and was included in this study for this reason, despite EHV-1 being non-zoonotic. The neuropathogenic genotype of EHV-1 (possessing the G_2254_ substitution in ORF30) was not detected in the sequence analysis of EHV-1 positive isolates in this study. A previous study in Australia detected the G_2254_ substitution in only a low percentage (1.5%) of isolates from abortion cases [87]. Neuropathogenic EHV-1 has been reported at a higher prevalence in abortion cases from other parts of the world, including 8.9% in Central Kentucky of the USA [88], 10.6% in Germany [89], and 25.9% in France [90]. These studies suggest that the prevalence of neuropathogenic EHV-1 in abortion cases may be increasing in different parts of the world, along with an increasing incidence of neurologic disease (equine herpesvirus myeloencephalopathy, EHM). The frequency of EHM in Australia is low compared to other countries which is likely associated with the low prevalence of ORF30 G_2254_ genotype in Australia [91].

Nugent et al., (2006), identified six different ORF68 groups for the first time using SNPs present in 559 bp region of ORF68 gene and proposed that this region as a suitable marker to study the geographical origin of the EHV [75]. In the current study, 83.33% (15/18) of the Australian EHV-1 isolates belonged to group 2 and this is consistent with that previously found for Australian EHV-1 isolates [87].

This study tested swabs taken from foetal tissues, rather than testing foetal tissues directly, in order to facilitate high throughput testing and to avoid the presence of PCR inhibitors in tissue extracts. PCR detection of pathogen DNA in tissue extracts can be limited by naturally occurring inhibitory substances [92, 93]. The inhibitor-containing substrate may be avoided by using a swab-transfer methods [94]. This study used rayon swabs which are not associated with PCR inhibition [95]. It is possible that the sensitivity of the assays was too low to detect pathogens if they were present at low copy numbers in the tested samples. It is also possible that pathogen detection may have been impacted by freeze/thawing of samples which can degrade DNA. Some of the tissues in this study had been in our archives for up to 25 years and may have experienced freeze/thawing over this time, although this is avoided whenever possible. DNA sizes smaller than 100 kb are less sensitive to freeze/thaw degradation. In this study the PCR product sizes were small (Table 2) which suggests that multiple freeze thaw cycles would have a small effect on detection [96].

In conclusion, this study revealed the presence of the zoonotic agent *C*. *burnetii* in aborted equine foetal tissues in Australia. *C*. *burnetii* was detected at a prevalence similar to EHV-1 (4% and 3%, respectively), however the low copy numbers of *C*. *burnetii* could suggest that the bacteria were not the cause of the abortion event. Future studies to assess any pathological changes associated with the presence of the bacteria are indicated, as are studies to determine the prevalence and load of C. *burnetii* in placental tissues from healthy foals. Together such studies will help to clarify the role of C. *burnetii* in equine abortion in Australia. The dose of *C*. *burnetii* for human infection is very low (only 10–15 organisms) [97], and therefore people handling material associated with equine abortions may be at risk of being infected with *C*. *burnetii*. This is in addition to risks posed by possible infection with *C*. *psittaci* [98]. Thus, it is recommended that appropriate measures to minimise the risk of infection, such as the use of personal protection equipment, vaccination and biosecurity procedures, be considered when handling aborted equine materials.

## Supporting information

S1 Table List of primers and probes used in this study. (DOCX)
