# Supplementary material for: Detection of Coxiella burnetii and equine herpesvirus 1, but not Leptospira spp. or Toxoplasma gondii, in cases of equine abortion in Australia - a 25 year retrospective study
Source: PLoS One. 2020 May 26;15(5):e0233100. doi: 10.1371/journal.pone.0233100 (PMC7250447; doi:10.1371/journal.pone.0233100)
Supplement: S1 Table — (DOCX) [file pone.0233100.s001.docx]

**S1 Table. List of primers and probes used in this study**

| Pathogen | Primers and Probes | Sequences (5’-3’) | Product size (bp) | References |
| --- | --- | --- | --- | --- |
| *C. burnetii* | *ompA-F* | CAGAGCCGGGAGTCAAGCT | 82 | (70) |
|  | *ompA-R* | CTGAGTAGGAGATTTGAATCGC |  |  |
| *Leptospira* spp. | *LipL32*-45F | AAGCATTACCGCTTGTGGTG | 242 | (71) |
|  | *LipL32*-286R | GAACTCCCATTTCAGCGATT |  |  |
|  | *LipL32*-189P | FAM-AAAGCCAGGACAAGCGCCG-BHQ1 |  |  |
| *T. gondii* | Toxo-F | AGAGACACCGGAATGCGATCT | 529 | (72) |
|  | Toxo-R | CCCTCTTCTCCACTCTTCAATTCT |  |  |
|  | Toxo probe | 56-FAM-ACGCTTTCCTCGTGGTGATGGCG‐IBFQ |  |  |
| *Herpesviruses* | DFA-F (1^st^ round) | GAYTTYGCNAGYYTNTAYCC | 415 | (73) |
|  | ILK-F (1^st^ round) | TCCTGGACAAGCAGCARNYSGCNMTNAA |  |  |
|  | KGI-R (1^st^ round) | GTCTTGCTCACCAGNTCNACNCCYTT |  |  |
|  | TGV-F (2^nd^ round) | TGTAACTCGGTGTAYGGNTTYACNGGNGT |  |  |
|  | IYG-R (2^nd^ round) | CACAGAGTCCGTRTCNCCRTADAT |  |  |
| EHV-1 ORF30 and ORF68 | ORF30.FL-F | ATCTCAGCTTTGATGGGGAG | 3663 | (74, 75) |
|  | ORF30.FL-R | AAAAGGAAACCATTCGCACT |  |  |
|  | ORF30-F1 | GCGCTACTTCTGAAAACG | 645 |  |
|  | ORF30-R1 | CCACAAACTTGATAAACACG |  |  |
|  | ORF68-F | TTG GCA TCT GAA CCG CTT GG | 764 |  |
|  | ORF68-R | AGA GTA GGC GTT CCA TCC AC |  |  |
